# Strategies of preserving genetic diversity while maximizing genetic response from implementing genomic selection in pulse breeding programs

**DOI:** 10.1007/s00122-022-04071-6

**Published:** 2022-03-22

**Authors:** Yongjun Li, Sukhjiwan Kaur, Luke W. Pembleton, Hossein Valipour-Kahrood, Garry M. Rosewarne, Hans D. Daetwyler

**Affiliations:** 1 Agriculture Victoria, AgriBio, Centre for AgriBiosciences, Bundoora, VIC 3083 Australia; 2 Agriculture Victoria, Grains Innovation Park, Horsham, VIC 3400 Australia; 3 School of Applied Systems Biology, La Trobe University, Bundoora, VIC 3083 Australia

## Abstract

**Key message:**

Genomic selection maximizes genetic gain by recycling parents to germplasm pool earlier and preserves genetic diversity by restricting the number of fixed alleles and the relationship in pulse breeding programs.

**Abstract:**

Using a stochastic computer simulation, we investigated the benefit of optimization strategies in the context of genomic selection (GS) for pulse breeding programs. We simulated GS for moderately complex to highly complex traits such as disease resistance, grain weight and grain yield in multiple environments with a high level of genotype-by-environment interaction for grain yield. GS led to higher genetic gain per unit of time and higher genetic diversity loss than phenotypic selection by shortening the breeding cycle time. The genetic gain obtained from selecting the segregating parents early in the breeding cycle (at F_1_ or F_2_ stages) was substantially higher than selecting at later stages even though prediction accuracy was moderate. Increasing the number of F_1_ intercross (F_1i_) families and keeping the total number of progeny of F_1i_ families constant, we observed a decrease in genetic gain and increase in genetic diversity, whereas increasing the number of progeny per F_1i_ family while keeping a constant number of F_1i_ families increased the rate of genetic gain and had higher genetic diversity loss per unit of time. Adding 50 F_2_ family phenotypes to the training population increased the accuracy of genomic breeding values (GEBVs) and genetic gain per year and decreased the rate of genetic diversity loss. Genetic diversity could be preserved by applying a strategy that restricted both the percentage of alleles fixed and the average relationship of the group of selected parents to preserve long-term genetic improvement in the pulse breeding program.

**Supplementary Information:**

The online version contains supplementary material available at 10.1007/s00122-022-04071-6.

## Introduction

Pulses including lentil (*Lens culinaris ssp. culinaris*), chickpea (*Cicer arietinum*), faba bean (*Vicia faba*), field pea (*Pisum sativum*), lupin (*Lupinus*) and mungbean (*Vigna radiata*) are grain legumes that are mainly produced for human consumption. These crops have been commercially grown in Australia since the 1970s, in rotation with cereals due to their ability to fix atmospheric nitrogen and management of weeds. Pulse crops are now mainstream in Australia’s cropping rotations and provide a valuable and sustainable source of protein (https://www.pulseaus.com.au). Australian pulses are in demand around the world for their quality, versatility and nutritional attributes (https://www.aegic.org.au/australian-grains/pulses/). Most pulses produced in Australia are exported to countries around the world, holding an enviable reputation for quality in global markets. Pulse breeding in Australia aims to increase high-quality grain production through improving regional adaptation, yield potential, disease resistance, and tolerance to harsh environments (GRDC 2019).

Genomic selection (GS) is an efficient selection method for improving quantitative traits in plant and livestock breeding by using high-density genome-wide markers often obtained by genotyping-by-sequencing (Bernardo and Yu 2007; Crossa et al. 2010; Meuwissen et al. 2001). The accuracy of genomic breeding values (GEBVs) depends on the size of the training population, the trait heritability, the diversity of the population (including their relatedness), and the density and distribution of genomic markers across the genome (Daetwyler et al. 2008; Erbe et al. 2013; Goddard 2009). Large training populations that capture a high proportion of the genetic variance with densely distributed genetic markers along the genome lead to more accurate estimations of genomic breeding values. To implement genomic selection, two groups of individuals are needed: the training population (genotyped and phenotyped) and selection candidates (often only genotyped). One of the benefits of GS is that the genomic breeding values of selection candidates can be estimated before their phenotypic performance is available. Selection can be conducted as soon as seeds are available; therefore, generation intervals (breeding cycle time) can be significantly shortened to increase genetic gain per unit of time. GS has been applied in pulse breeding programs for selecting phenological traits, grain yield traits and disease resistance (Carpenter et al. 2018; Haile et al. 2019; Li et al. 2017a; Roorkiwal et al. 2018, 2020; Tayeh et al. 2015).

Preservation of genetic diversity in a breeding population is also essential to obtain genetic improvement in the longer term (Goddard 2009; Li et al. 2008). In plant breeding, usually a small number of individuals are selected as parents of the next generation, which causes loss of some alleles. Selection based on breeding values increases the probability of selecting related individuals as compared to phenotype-based selection. GS tends to accelerate the process of loss of genetic diversity in a population, especially per unit of time when coupled with high selection intensity. It could potentially double the speed at which genetic variation is lost within livestock breeds (Kristensen et al. 2015; Pedersen et al. 2009; Pertoldi et al. 2014). Two main reasons that cause loss of genetic variation in the breeding populations include the loss or fixation of favourable quantitative trait loci (QTL) alleles and the increased relatedness between selected individuals, which reduces trait variation and, in turn, reduces long-term genetic response (Falconer and Mackay 1996; Jannink 2010; Li et al. 2008). Several strategies of preserving genetic variation have been applied in livestock and plant breeding, including minimizing the average coancestry (Cervantes et al. 2016; Hallander and Waldmann 2009; Meuwissen 1997; Villanueva et al. 2006), minimizing inbreeding coefficients (Brisbane and Gibson 1995; Li et al. 2008; Lin et al. 2017; Meuwissen 1997), avoiding the selection of closely related individuals (Lindgren and Mullin 1997), and reducing the loss of favourable QTL alleles (Vanavermaete et al. 2020). In recent years, the genomic relationship matrix has been used to control inbreeding levels and maximizing long-term genetic gain (De Beukelaer et al. 2017; Lin et al. 2017; Pryce et al. 2012; Santantonio and Robbins 2020; Sonesson et al. 2012).

Plant breeding strategies can be evaluated using computer simulations as well as long-term performance and consequences can be predicted without field experiments that are labour-intensive and time-consuming (Li et al. 2012). The essence of computer simulations is to sample as many conditions as possible that might be encountered in breeding practice. The simulations can provide insights into the identification of best strategies for maximizing genetic gain, preserving genetic diversity, and optimizing operational costs by shortening generation intervals, optimizing parental crosses, and introducing trait variations from external resources. Two types of simulations are used in plant breeding strategies; deterministic and stochastic. Deterministic simulations are designed to capture underlying mechanism or a natural process based on equations without random variables and degree of randomness (Hahl and Kremling 2016), while stochastic simulations are used to mimic entire populations under selection, for multiple breeding cycles (Liu et al. 2019; Pedersen et al. 2009).

The objectives of this study were to investigate strategies for implementing GS in a lentil breeding program using a stochastic computer simulation. Using phenotypic selection (conventional breeding) as a benchmark, we investigated: cycling parents back to the germplasm pool from different stages of the breeding cycle; testing the effects of F_1_ intercross (F_1i_) family number and size on genetic gain and genetic diversity loss per unit of time; and preserving genetic diversity while maximizing genetic gain.

## Materials and methods

### Breeding programs

**Fig. 1:**
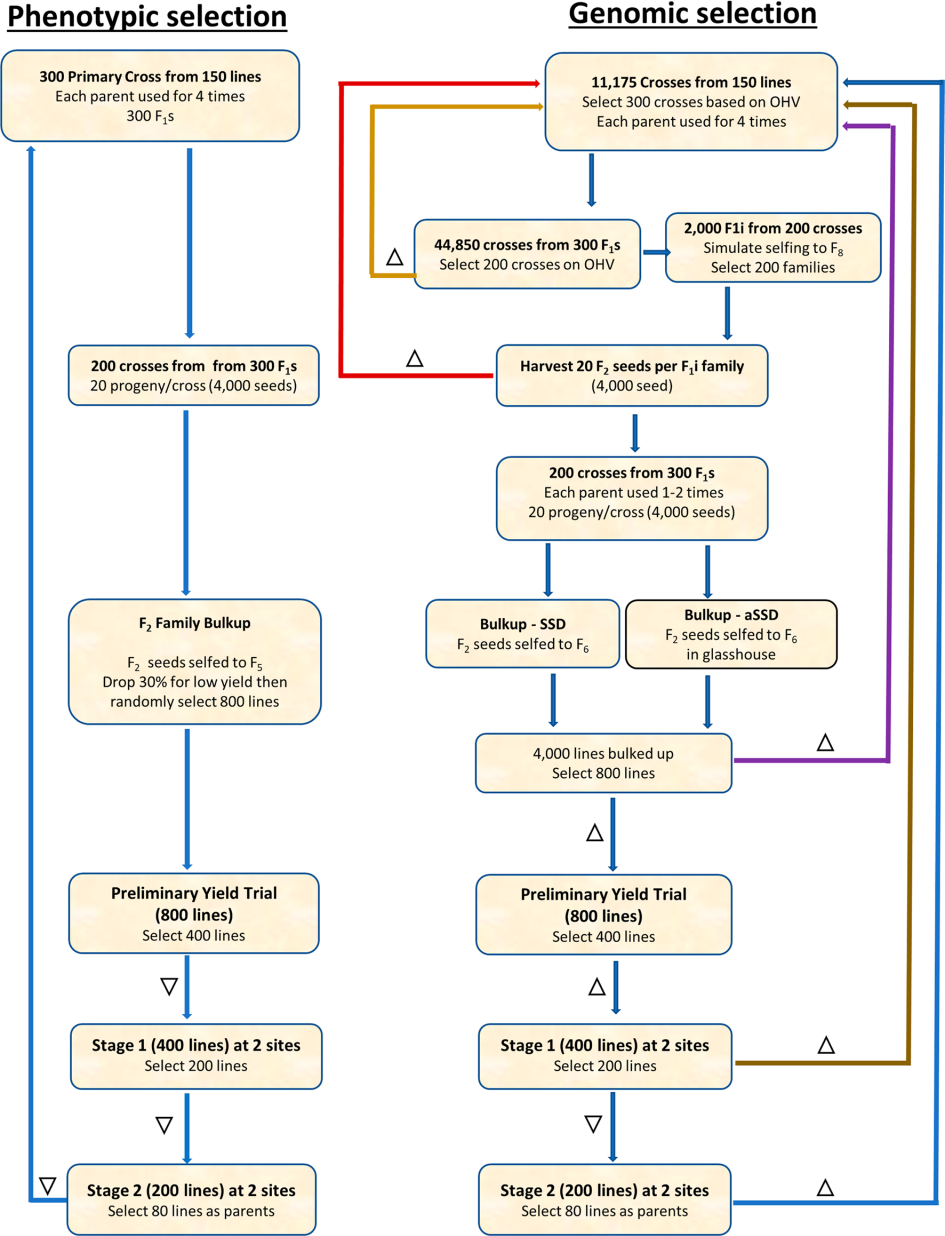
Diagram of simulated pulse breeding programs for phenotypic selection and genomic selection. $\nabla$: selection on phenotypic selection, ∆: selection on the genomic breeding values, SSD: single seed descent, and aSSD: accelerated single seed descent

This simulation modelled a lentil breeding program with two main pathways phenotypic selection (PS) and genomic selection (GS). It consisted of 6 main steps: primary crosses, F_2_ selfing in PS or intercrossing in GS, bulk-up (F_3_–F_5_ in PS or F_3_–F_6_ in GS), preliminary yield trial (PYT), Stage 1 trials, and Stage 2 trials (evaluation of advanced breeding lines) (Fig. 1). In the PS pathway, 300 primary crosses (F_1_ progeny) were generated from 150 inbred lines by random mating, where each inbred line was used up to 4 times. Three hundred F_1_ families were randomly selected with 20 F_2_ seeds per family. These F_2_ seeds were bulked up within F_1_ families until F_5_ (family-bulkup). Thirty per cent of single F_5_ plants were dropped based on low seed yield, and 800 lines of the remaining 70% were then randomly selected for the PYT stage. These selection pressures mimicked selection applied during bulk-up for a variety of breeder’s preferred traits with some impact on yield. In the PYT, lines were tested in a partial replication trial for disease resistance, grain weight, and grain yield, of which 400 lines were further selected based on phenotypic performance. In Stage 1, 400 lines were tested in a trial with 2 replicates at two environments and assessed for disease resistance, grain weight and grain yield, with the top-performing 200 lines being selected based on phenotypic performance. In Stage 2, 200 lines were tested in a trial with 2 replicates at two environments and assessed for disease resistance, grain weight, and grain yield, and 80 lines were selected based on phenotypic performance and used as the parents of the next crossing cycle.

In the GS pathway, 300 primary crosses and 200 F_1_ intercrosses (F_1i_) were selected based on the optimal haploid value (OHV) (Daetwyler et al. 2015). The OHV of F_1_ was simulated from all possible crosses that could be made using 150 inbred parents. A selection index of OHVs was calculated using 30% disease resistance, 30% grain weight, and 40% grain yield. Out of all possible crosses, the top 300 crosses were selected, restricting the use of each inbred line to up to 4 times to generate 300 F_1_s. These 300 F_1_s were then used to model F_1i_ intercrosses (crossing F_1_s to each other; 4-way crosses). All possible crosses were simulated from 300 F_1_s and their OHVs were calculated. Two hundred F_1i_s with the highest mean OHVs were selected. Each F_1_ intercross produced 10 seeds resulting in 2000 F_1i_s in total. These 2000 F_1i_s were simulated in silico for selfing to F_8_ through single seed descent (SSD) where 20 seeds were used at the F_8_ stage. The 200 F_1i_s with the highest mean OHVs plus the standard deviation were selected for selfing. Twenty F_2_ seeds were harvested from each F_1i_; therefore, a total of 4000 seeds were available for the bulk-up step with two options: (a) single seed descent (SSD) with each F_1i_ seed selfed to F_6_ (4 years in the field), (b) accelerated single seed descend (aSSD) with each F_1i_ seed selfed to F_6_ in a glasshouse (1.5 years) to reduce the time for selfing. At the end of the bulk-up stage, 4000 lines were genotyped and based on GEBVs, 800 were selected for evaluation at the next stage (PYT). PYT along with subsequent stages i.e. Stage 1 and Stage 2 had the same trial settings as the phenotypic selection with the only difference being that genomic selection was applied at PYT and Stage 2.

Parents of each new breeding cycle were selected from a germplasm pool. The germplasm pool consisted of 1568 lentil inbred lines that were used as the reference set in this study (details given below). Eighty inbred lines were selected from each breeding cycle and added to the germplasm pool. A hundred fifty parents initiating the primary crosses were selected from the germplasm pool, based on phenotypic performance (in PS) or GEBVs (in GS). The new inbred lines that were added to the germplasm pool were selected from Stage 2 in PS. In GS, three parental selection time points from F_6_, F_2_ and F_1_ were compared to investigate the effect of shortening the breeding cycle on the rate of genetic gain.

### Genomic data of lentil inbred lines

Transcriptome genotyping-by-sequencing (GBS) data from 1,568 lentil inbred lines (the base population) genotyped at Agriculture Victoria with 88,376 single nucleotide polymorphisms (SNPs) formed the base generation for the simulations. Missing genotypes were imputed using LinkImpute (Money et al. 2015). After removing SNPs with a call rate below 0.5 and a minor allele frequency < 0.01, 63,967 SNPs remained of which 30,000 were randomly chosen. There were between 3200–5200 SNPs on each of the seven chromosomes. The genetic map length of seven chromosomes ranged from 192.7 centimorgans to 429.7 centimorgans (Sudheesh et al. 2016).

### Simulation

In this study, three traits, i.e. disease resistance, grain weight and grain yield, with an assumption of narrow-sense heritabilities of 0.5, 0.75 and 0.25, respectively, were simulated at two environments. Only additive gene action was simulated, and each trait was assumed to be controlled by approximately 1000 QTL. Seventy per cent of QTL were randomly assigned being overlapped between traits within an environment and between environments within a trait. QTL effects were sampled from a normal distribution with a mean of zero and a standard deviation of 1. Correlations of QTL between traits were zero whereas between environments was 0.8 for disease resistance and grain weight (low to moderate level of genotype by environment (G × E) interactions) and 0.4 for grain yield (a high level of G × E interaction) (Li et al. 2017b).

The true breeding value (TBV) of an individual ($g$) was calculated as the sum of the individual QTL additive effects using an equation $g=Ma$, where $M$ is the incidence matrix related to the number of QTL alleles (0, 1, or 2) and $a$ is a vector of the allele substitution effects of QTL. Phenotype of a trait was the sum of the true breeding value and random residual that was sampled from a normal distribution with a mean of 0 and a variance of $\sqrt{\frac{1-{h}^{2}}{{h}^{2}}{\sigma }_{g}^{2}}$, where ${h}^{2}$ is the heritability of trait and ${\sigma }_{g}^{2}$ is the genetic variance.

Recombinations and mutations were simulated when gametes were generated. Recombinations were sampled per chromosome from a Poisson distribution with lambda equal to 1. Mutations were sampled from a uniform distribution with a mutation rate of 0.001.

### GEBV and OHV estimation

GEBVs were estimated using a two-step approach. Firstly, marker effects were estimated using Bayesian ridge regression based on phenotypes and genotypes of the training population. The training population consisted of all individuals from Stage 2 (400 individuals per cycle) across the three most recent breeding cycles. GEBVs of individuals for traits under study were calculated based on the marker effects and their genotypic profiles. Bayesian ridge regression was implemented using R package BGLR (de los Campos and Pérez 2013) with the following Eq. (1):

1 $${\varvec{y}}=1{\varvec{\mu}}+{\varvec{X}}{\varvec{\beta}}+{\varvec{u}}+{\varvec{\varepsilon}}$$

where ${\varvec{y}}$ is a vector of phenotypes, $\mu$ is an intercept, ${\varvec{X}}$ is an incidence matrix that links phenotypes to ${\varvec{\beta}}$ the vector of marker substitution effects, ${\varvec{u}}$ is a vector of random effects, and ${\varvec{\varepsilon}}$ is a vector of the residuals. GEBVs ($\widehat{{\varvec{g}}}$) were predicted as the linear combination of the marker effects as Eq. (2):

2 $$\widehat{{\varvec{g}}} = {\varvec{X}}^{\prime} {\varvec{\beta}}$$

where $\it X'$ contains the genotypes of selection candidates. The OHV was estimated with five OHV segments on each chromosome (Daetwyler et al. 2015). For each OHV segment, a haplotype value ($HV$) was calculated for both haplotypes with Eq. (3):

3 $$\mathrm{HV}=\sum_{k=1}^{m}{h}_{k}{\beta }_{k}$$

where $m$ is the total number of marker loci in the OHV segment, $k$ is the locus within the OHV segment, and ${\beta }_{k}$ is the marker allele substitution effect at locus *k* estimated from Eq. (1). The OHV is the sum of the higher *HV* of each segment across all chromosomes.

### Evaluation of the breeding program

The simulation was conducted for eight breeding cycles with 50 replicates for each scenario (defined below) and 150 parents at the beginning of breeding cycle 1 were resampled from the base population in each replicate. PS was conducted at breeding cycles 1–8 and GS was divergently conducted at breeding cycles 4–8, using the end of phenotypic cycle 3 as the starting point. Selection was conducted using a selection index (${I}_{\mathrm{GEBV}}$) in Eq. (4):

4 $$I_{{{\text{GEBV}}}} = w_{D} \times \frac{{u_{D} }}{{s_{{u_{D} }} }} + w_{W} \times \frac{{u_{W} }}{{s_{{u_{W} }} }} + w_{Y} \times \frac{{u_{Y} }}{{s_{{u_{Y} }} }},$$

where ${w}_{D}$, ${w}_{W}$ and ${w}_{Y}$ are index weights, ${u}_{D}$, ${u}_{W}$ and ${u}_{Y}$ are GEBVs in the genomic selection or phenotypic values in the phenotypic selection, ${s}_{{u}_{D}}$, ${s}_{{u}_{W}}$ and ${s}_{{u}_{Y}}$ are the standard deviations of GEBVs or phenotypic values for disease resistance, grain weight and grain yield, respectively. Index weights ${w}_{D}$, ${w}_{W}$ and ${w}_{Y}$ had values of 0.3, 0.3 and 0.4, respectively, in both phenotypic selection and genomic selection.

Genetic gain and genetic diversity achieved in a breeding program as well as predictive accuracy of the GEBVs were evaluated. The genetic gain was defined as the average true breeding values of individuals, expressed in units of the additive genetic standard deviations of the lentil inbred line base population. An aggregate genetic gain was calculated as the sum of genetic gain of disease resistance, grain weight and grain yield weighted by their selection index weights.

The level of genetic diversity observed in a scenario was evaluated using TBV variances of traits, the number of marker alleles fixed (NAF) and the average genomic relationship of parental lines that were used to generate primary crosses at the beginning of each breeding cycle. An aggregate TBV variance was also calculated as the sum of TBV variance of disease resistance, grain weight and grain yield weighted by their selection index weights. The average genomic relationship was expressed as the mean off-diagonal of the genomic relationship matrix (GRM). The genomic relationship matrix was derived using the method proposed by VanRaden (2008). Higher TBV variances, lower NAF and lower average relationships imply higher genetic diversity.

The prediction accuracy of GEBVs was defined as the correlation between GEBVs and TBV. When evaluating the prediction accuracy of GEBVs, phenotypes of individuals to be ranked were not used in estimating marker effects, which means that GEBVs were calculated using marker effects estimated at the previous breeding cycle.

### Simulation scenarios

#### Length of generation interval

Scenarios for shortening generation intervals with phenotypic selection and genomic selection were investigated (Table 1). GS scenario tested different options for seed bulk-up, i.e. single-seed-descent (SSD) versus accelerated single-seed-descent (aSSD) during generations of F_3_-F_6_, and different stages, i.e. Stage 2 (STG2), F_6_, F_2_ or F_1_ for cycling parents into crossing.

**Table 1:** Scenarios in phenotypic selection (PS) and genomic selection (GS) with parents selected from Stage 2, F_6_, F_2_ or F_1_ in three bulking-up methods in F_3_–F_6_ [family-bulk-up, single-seed-descent (SSD) or accelerated single-seed-descent in the glasshouse (aSSD)] with or without phenotyping of F_2_ families and diversity preservation

Scenario	Selection method	Bulk-up method (F_3_–F_6_)	Parents selected from	Generation interval (years)
PS_STG2	PS	Family-bulkup^†^	Stage 2	8
GS_SSD_STG2	GS	SSD	Stage 2	8.5
GS_SSD_F_6_	GS	SSD	F_6_	5
GS_SSD_F_2_	GS	SSD	F_2_	1
GS_SSD_F_1_	GS	SSD	F_1_	0.5
GS_aSSD_STG2^ǂ^	GS	aSSD	Stage 2	6
GS_aSSD_F_6_	GS	aSSD	F_6_	2.5
GS_aSSD_F_2_^ξǂ^	GS	aSSD	F_2_	1
GS_aSSD_F_1_	GS	aSSD	F_1_	0.5

^ξ^Additional option was applied where 50 F_2_ families were planted in the field and their phenotypes of disease resistance, grain weight and grain yield were added to the training population (GS_aSSD_F_2__PH)

^ǂ^Additional option was applied where diversity preservation strategy was applied (GS_aSSD_STG2_DP and GS_aSSD_F_2__DP)

^†^Family-bulk-up was conducted from F_3_–F_5_

#### F_1i_ family sizes

In the genomic selection scenario with accelerated single seed descent (aSSD), the effects of F_1i_ family size on genetic gain and genetic diversity were investigated using scenarios defined by a different number of F_1i_ families, i.e. 100, 200 and 400 to generate a final constant number of 4000 seeds. Other scenarios with a different number of seeds per F_1i_ family, i.e. 20, 40 or 80 to make 4000, 8000 or 16000 seeds from 200 F_1i_ families were also simulated.

#### Adding phenotypes of F_2_ families to the training population

The training population consisted of inbred lines chosen from the three most recent breeding cycles, 400 inbred lines per breeding cycle randomly chosen from Stage 2. In the accelerated scenarios that selected parents at F_2_, the selection candidates become further remote from the training population due to a reduced cycle time. Therefore, a choice of phenotyping 50 F_2_ families was also included to add phenotypes to the training population more quickly, where the average phenotypic performance and marker genotypic values across the individuals per F_2_ family were used as the equivalent of one individual in the training population.

#### Strategy to preserve diversity

To maintain more diversity in the GS scenario, restrictions on the average relationship of the 150 selected parents and the number of fixed alleles within those parents were implemented with a genetic algorithm (Holland 1992). The fitness function in genetic algorithms ($f$) is shown in Eq. (5):

5 $$f = I_{{{\text{GEBV}}}} - \lambda_{1} \overline{{{\text{REL}}}} - \lambda_{2} {\text{NAF}}$$

where ${I}_{\mathrm{GEBV}}$ is the standardized selection index calculated using Eq. (4), and $\overline{\mathrm{Rel}}$ is the average of the genomic relationship of selected individuals, *λ*_1_ is a penalty applied to the average relationship of selected individuals, $\mathrm{NAF}$ is the number of alleles fixed of selected individuals and, *λ*_2_ is a penalty applied to $\mathrm{NAF}$ of selected individuals. Diversity preservation strategies with *λ*_1_ = 1 and *λ*_2_ = 10^–6^ were compared with a scenario without penalties in aSSD where parents were selected from F_2_. The genetic algorithm was applied with a population size of 2000, with 1000 iterations, a mutation rate of 0.001 and 20% highest fitness subsets randomly crossed each with one point crossover. Penalties on *λ*_1_ and *λ*_2_ and parameters used in the genetic algorithm were defined by choosing from a range of arbitrary values with which the optimal output of genetic diversity and genetic gain were achieved.

## Results

### Length of generation interval

**Fig. 2:**
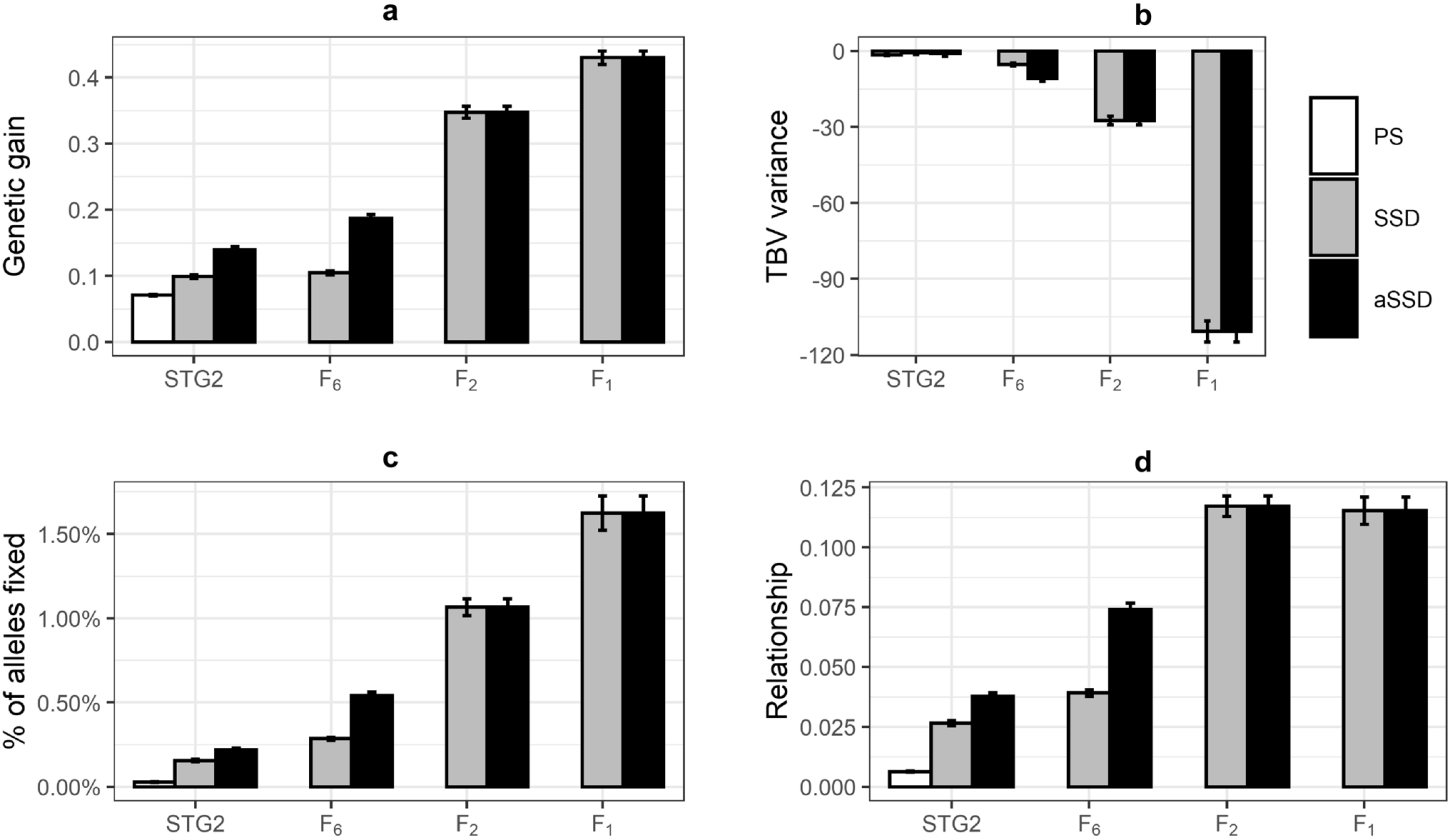
Mean annual aggregated genetic gain (**a**), TBV variance (**b**), percentage of alleles fixed (**c**) and the genomic relationship (**d**) achieved with parents selected from Stage 2 (STG2) with PS and STG2, F_6_, F_2_ or F_1_ with GS single-seed-descent (SSD) or accelerated SSD (aSSD)

All GS scenarios led to a higher rate of genetic gain when compared to PS (Fig. 2a, Suppl Figure 1). Simulations in each scenario was conducted for 5 breeding cycles; therefore, the SSD scenario with parents selected from STG2 lasted for 42.5 years, whereas it only lasted for 7.5 years when parents were selected from F_2_. A clear trend for increased genetic gain with decreased cycle time was observed from GS scenarios. GS increased genetic gain per year by 40% in SSD-STG2 scenario and 98% in aSSD-STG2 (with a family bulking-up in F_3_–F_6_) over PS. If parents were selected from F_6_ (at the end of bulking-up), GS increased genetic gain per year by 90% with SSD-F_6_ and 165% with aSSD-F_6_ over PS. No difference in genetic gain was observed between SSD and aSSD if parents were selected from F_2_ or F_1_ because SSD and aSSD were selected before bulk-up and with the same composition of the reference population. The additional genetic gain achieved in GS with SSD or aSSD with parents selected from F_2_ and F_1_ stage was 393% and 547%, respectively. Details of genetic gain achieved in each trait are given in Suppl Figure 1.

When selecting parents from STG2, GS scenarios led to a higher percentage of alleles fixed and higher genomic relationship than PS. Furthermore, GS with aSSD led to a higher percentage of alleles fixed and genomic relationship as compared to GS with SSD, while there was no difference in TBV variance decrease in PS versus GS as well as between GS with aSSD and GS with SSD (Fig. 2b–d and Suppl Figure 2). Selecting parents from F_6_ resulted in a greater TBV variance decrease, higher percentage of alleles fixed and higher genomic relationship than selecting parents from STG2. Reducing time taken for bulk-up from F_3_ to F_6_ in GS (aSSD) also increased TBV variance reduction, percentage of alleles fixed and genomic relationship than a normal bulk-up from F_3_ to F_6_ in GS (SSD).

As observed in the aggregate genetic gain, when parents were selected from F_1_ or F_2_, the bulk-up method (GS-SSD or GS-aSSD) from F_3_ to F_6_ did not affect the level of genetic diversity loss. Within GS-aSSD scenario, the reduction in TBV variance was nearly two-fold when parents were selected from F_2_ as compared to the reduction observed when parents were selected from F_6_. Furthermore, when parents were selected from F_1_, the reduction in TBV variance was nearly three-fold of the reduction observed when parents were selected from F_2_. Selecting parents from F_1_ and F_2_ doubled and tripled the percentage of alleles fixed respectively when compared to selecting parents from F_6_ in GS-aSSD. Selecting parents from F_2_ and/or F_1_ resulted in a similar level of genomic relationship increase of 160% of the level observed when selecting parents from F_6_ in GS with aSSD.

**Fig. 3:**
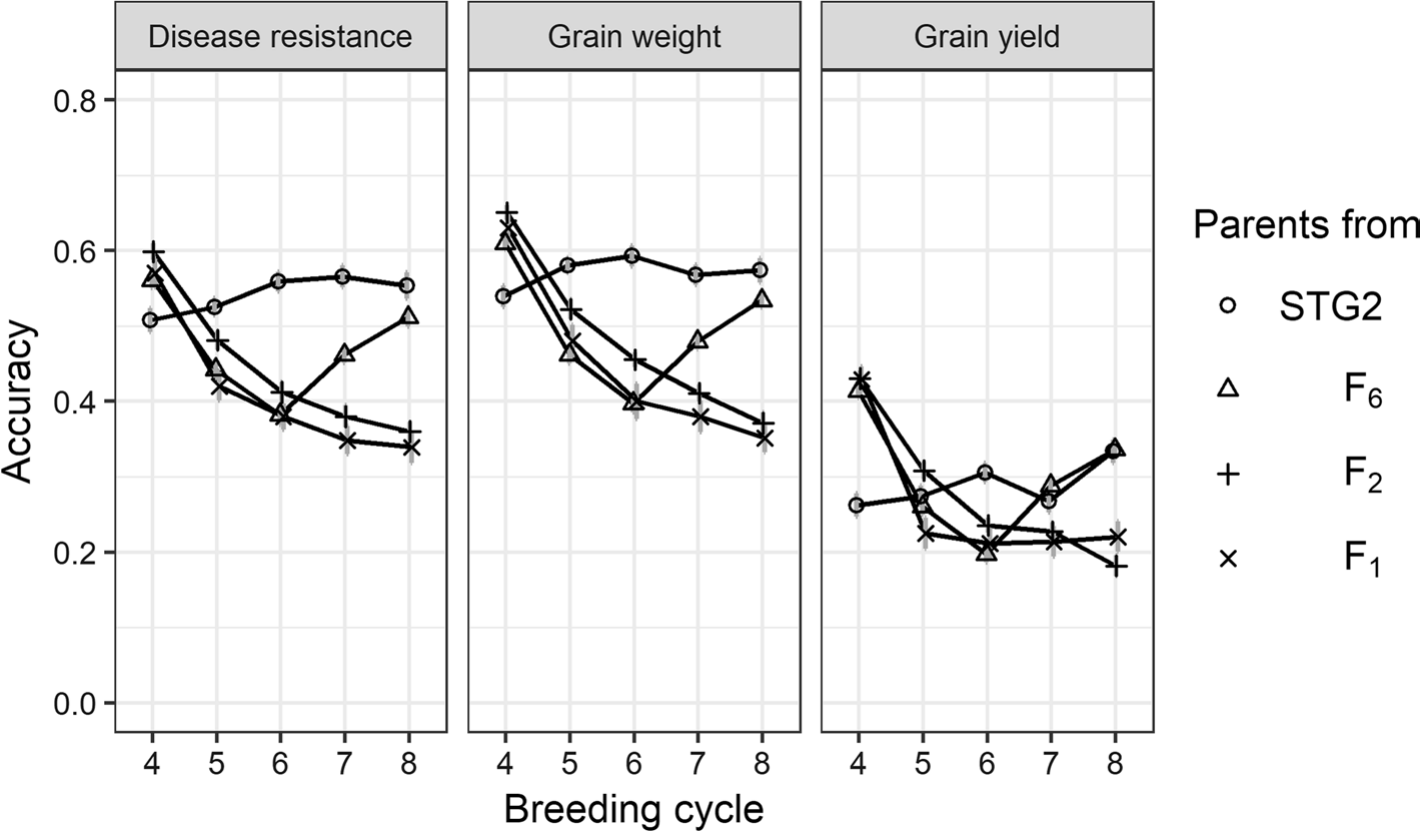
The accuracy of GEBVs for disease resistance, grain weight and grain yield in GS with single-seed-descend (SSD) in F_3_-F_6_ when parents were selected from Stage 2 (STG2), F_6_, F_2_ or F_1_

The accuracy of GEBVs for grain yield was lower than that for disease resistance and grain weight, as grain yield had low heritability and a high level of G × E interactions (Fig. 3). The accuracy for grain weight was slightly higher compared to disease resistance as the former had higher heritability. When parents were selected from STG2, phenotypes of the training population were updated every breeding cycle so that the accuracy of GEBVs for this scenario was increasing. When parents were selected from F_2_ and F_1_, the generation interval was reduced to 1 or 0.5 years, respectively, and phenotypes for estimating marker effects in breeding cycles 4 to 8 all came from breeding cycle 3. As a result, the accuracy of GEBVs decreased from breeding cycle 4 to breeding cycle 8. When parents were selected from F_6_, phenotypes generated at breeding cycle 4 were available for estimating marker effects at the end of breeding cycle 6; therefore, the accuracy of GEBVs increased from breeding cycle 7. When using the same training population, for example, at breeding cycle 4, the accuracy of GEBVs was the highest when parents were selected from F_2_ and the lowest when parents were selected from STG2.

### F_1i_ family size

**Fig. 4:**
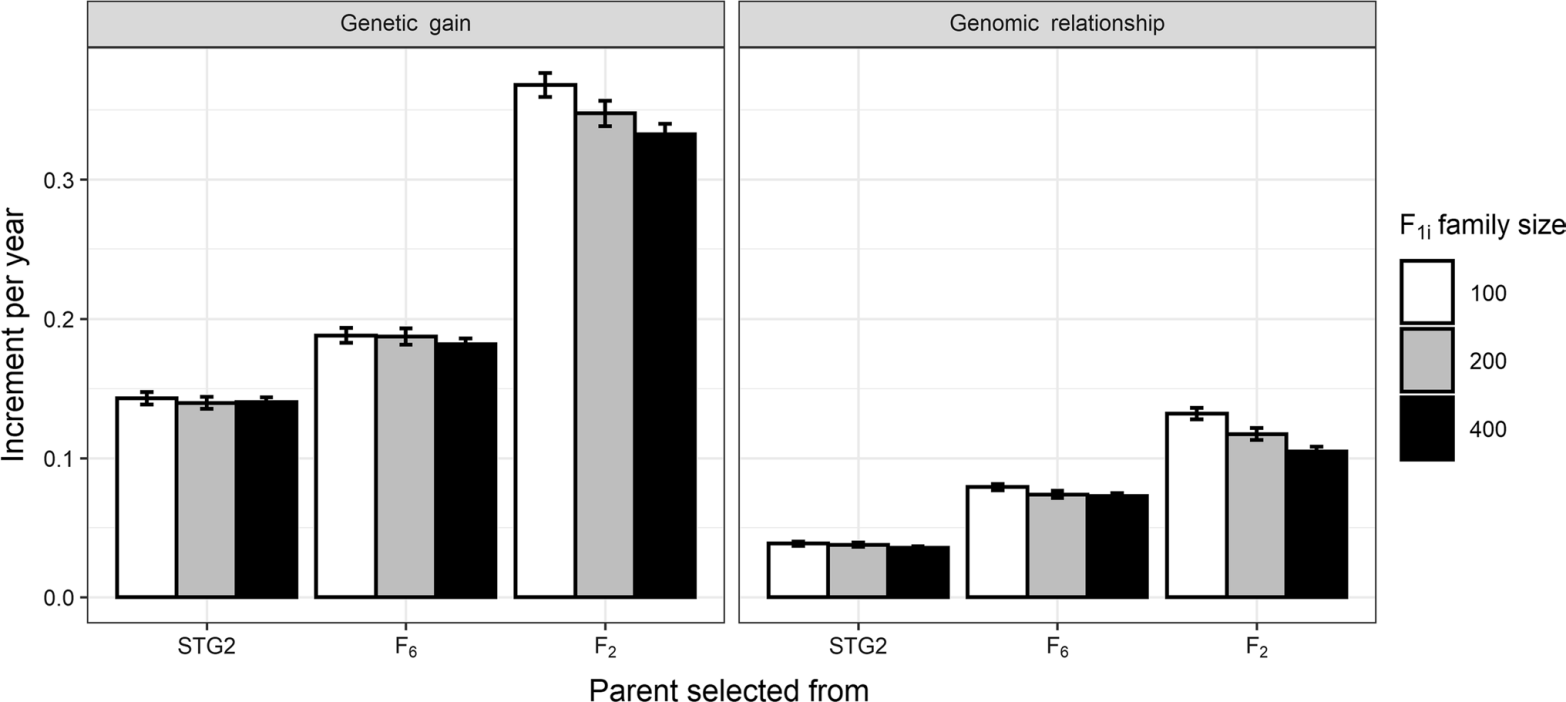
Mean annual aggregated genetic gain and the genomic relationship in GS with parents selected from Stage 2 (STG2), F_6_ or F_2_ for scenarios with 100, 200 and 400 F_1i_ families to make a constant number of 4000  seeds

Two scenarios were investigated: a constant (fixed) number of 4000 seeds (F_1i_ individuals) generated from a varying number of F_1i_ families (100, 200 and 400) and a varying number of seeds (20, 40 and 80) per F_1i_ family from a fixed number of F_1i_ families i.e. 200. For a constant number (*N* = 4000) of F_1i_ individuals, increasing the number of F_1i_ families and decreasing the number of seeds within family led to lower aggregated genetic gain and less genomic relationship per year (Fig. 4). Differences in the aggregated genetic gain and the aggregated loss of genetic diversity were observed among F_1i_ family sizes of 100, 200 and 400 with parents selected from F_2_. This is because F_1i_ from F_2_ selection had a larger variety of chromosome segments (i.e. increased diversity due to not crossing inbred lines) and having more progeny helps sort out the best GEBVs.

**Fig. 5:**
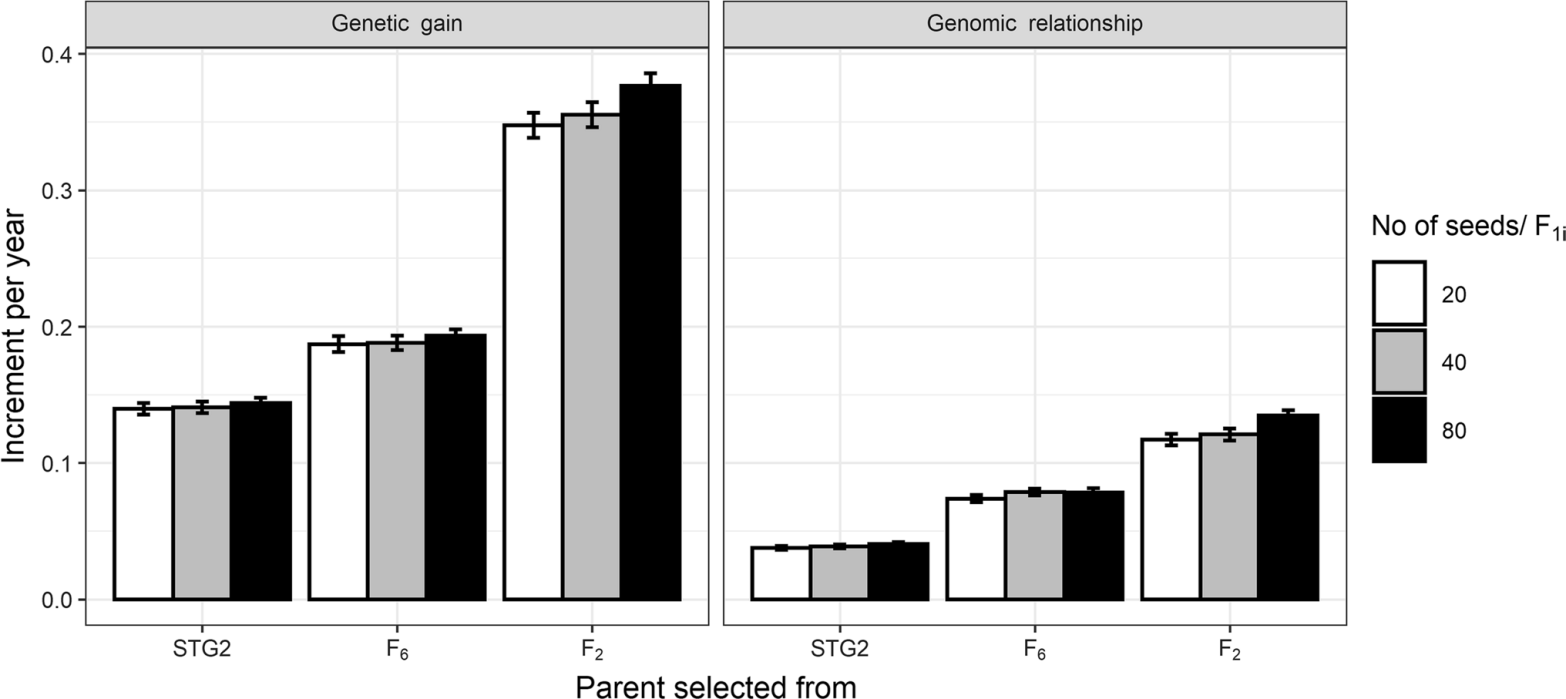
Mean annual aggregated genetic gain and the genomic relationship in GS with parents selected from Stage 2 (STG2), F_6_ or F_2_ for scenarios with different number of 20, 40 or 80 seeds per F_1i_ family to make 4000, 8000 or 16,000 seeds in total from a constant 200 F_1i_ families

For a constant number of F_1i_ families (*N* = 200), increasing the number of seeds from each F_1i_ led to higher aggregated genetic gain and higher loss of genetic diversity per year only when parents were selected at F_2_ (Fig. 5). This was likely due to higher selection intensity and the use of segregating parents leading to more diverse crosses.

### F_2_ phenotyping

**Fig. 6:**
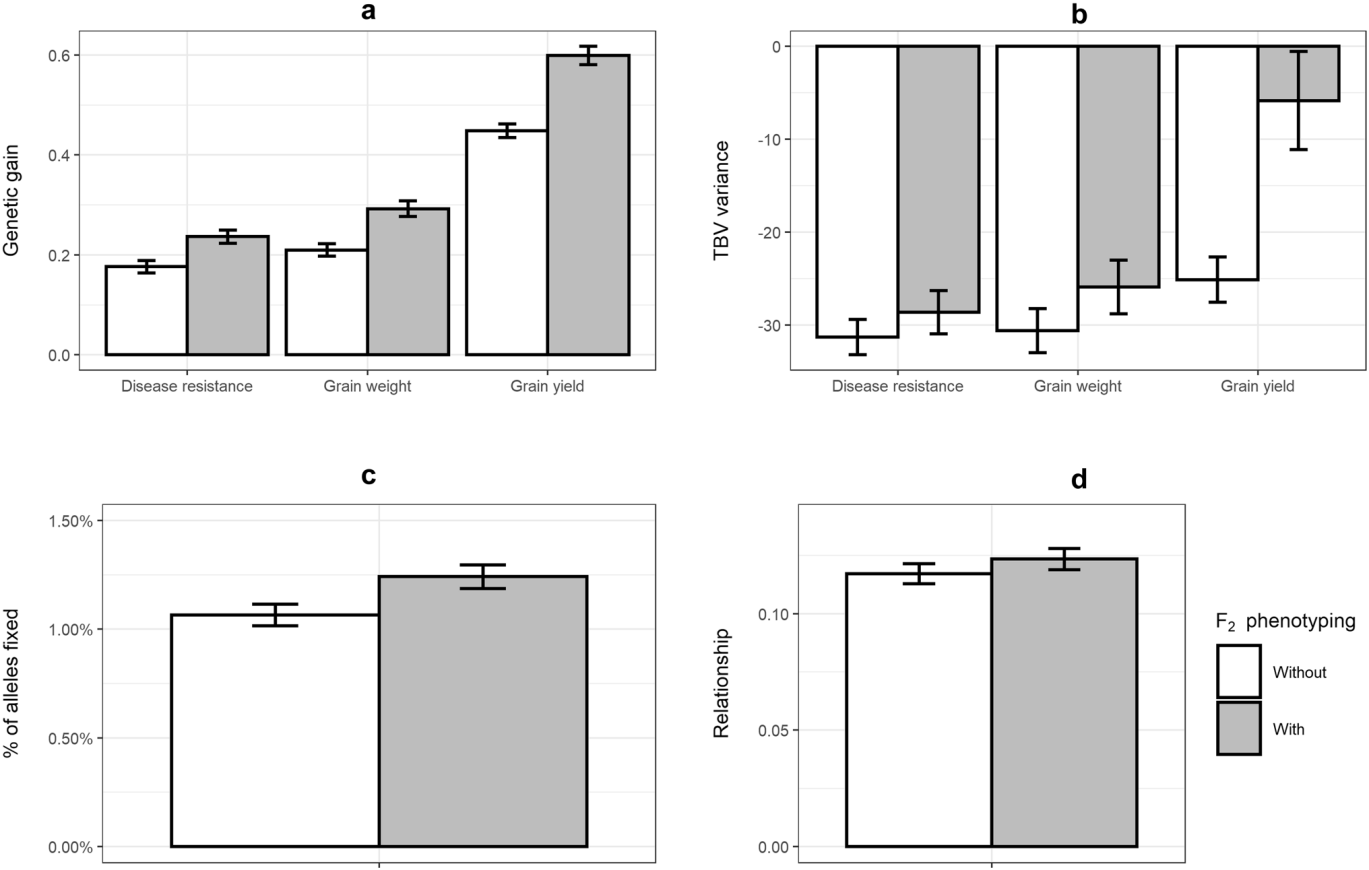
Mean annual changes of genetic gain (**a**) and TBV variance (**b**) in disease resistance, grain weight and grain yield, percentage of alleles fixed (**c**) and the genomic relationship (**d**) between scenarios with (grey bars, GS_aSSD_F_2__PH) or without (white bars, GS_aSSD_F_2_) additional phenotypes from F_2_ families in GS with aSSD in F_3_–F_6_ when parents were selected from F_2_

In scenario GS_aSSD_F_2__PH (genomic selection with the accelerated bulkup in glasshouse, parents selected from F_2_ and 50 F_2_ families being phenotyped), adding additional phenotypes obtained from F_2_ families to the training population significantly increased genetic gain for each trait (Fig. 6). When looking into the components of genetic diversity, adding additional phenotypes into the training population slowed down the TBV variance reduction, especially for grain yield, but increased the number of alleles fixed and the mean relationship between parents selected.

**Fig. 7:**
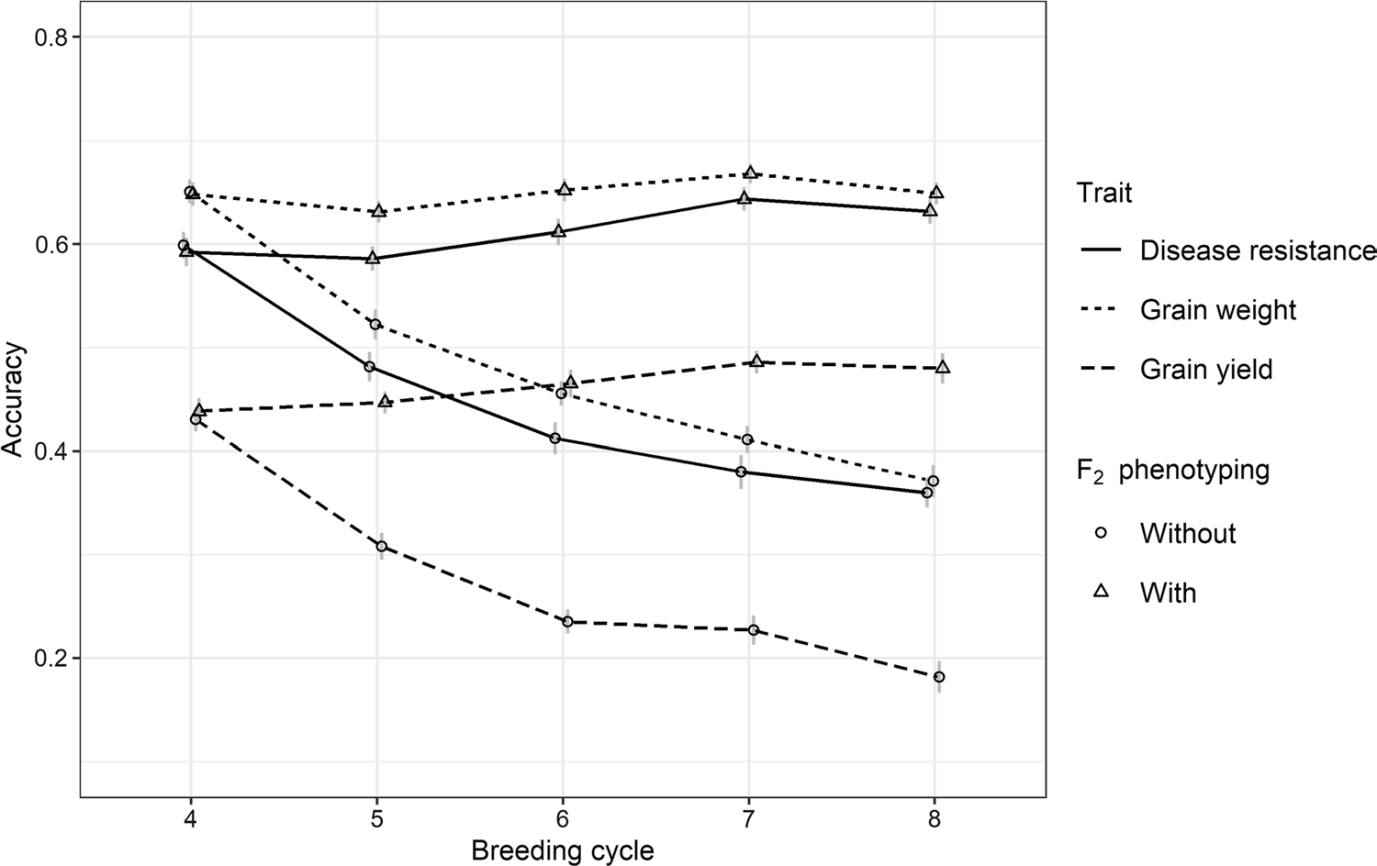
The accuracy of GEBVs for disease resistance, grain weight and grain yield in genomic selection with (GS_aSSD_F_2__PH) or without (GS_aSSD_F_2_) adding additional phenotypes from F_2_ families in GS with aSSD in F_3_–F_6_ when parents were selected from F_2_

The accuracy of GEBVs without adding F_2_ family phenotypes into the training population trended downwards because no new phenotypes were available from breeding cycle 4 to breeding cycle 8 (Fig. 7). With these new phenotypes available, the accuracy of GEBVs, the accuracy of GEBVs increased slightly over breeding cycles. The patterns of GEBV accuracy across traits were similar, with the highest for grain weight and the lowest for grain yield.

### Diversity preservation

**Fig. 8:**
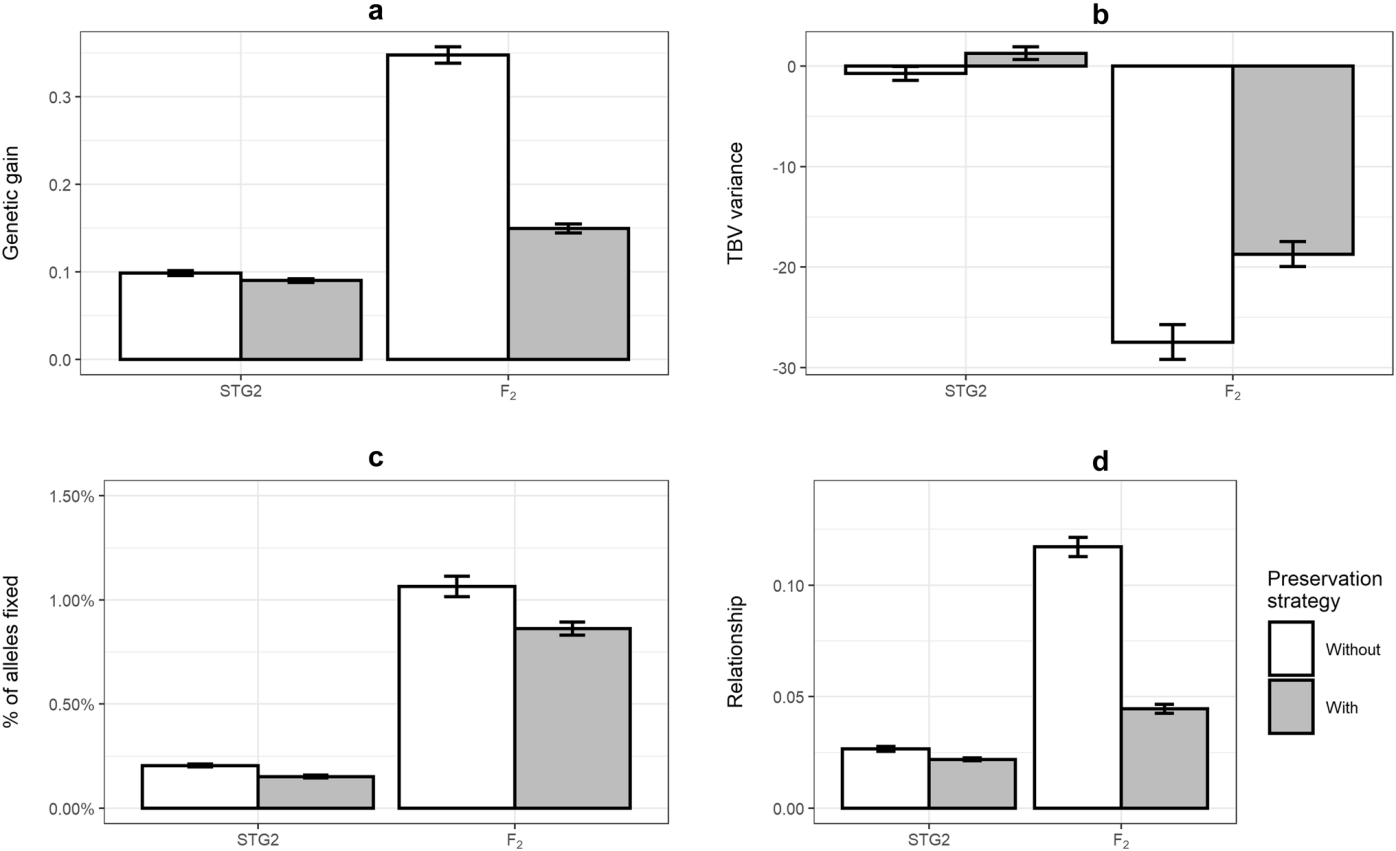
Mean annual changes of the aggregated genetic gain (**a**) and the aggregated TBV variance (**b**), percentage of alleles fixed (**c**) and the genomic relationship (**d**) between scenarios with (grey bars) or without (white bars) genetic diversity preservation strategy in GS with aSSD in F_3_–F_6_ when parents were selected from Stage 2 (STG2) and F_2_. Scenario with preservation strategy had a penalty on the genomic relationship (${\lambda}_{1}$=1) and a penalty on the number of alleles fixed (${\lambda_{2}}$=10^–6^)

A genetic diversity preservation strategy that restricted the number of alleles fixed as well as the average relationship between selected parents was applied to two scenarios where parents were selected from Stage 2 and F_2_ (GS_SSD_STG2_DP and GS_SSD_F_2__DP). The magnitude of genetic diversity loss per unit of time was reduced in terms of TBV variance reduction, percentage of fixed alleles and the average relationship among parents; however, the genetic gain was also reduced, compared to the scenarios without applying the genetic diversity preservation strategy (Fig. 8). In the case of parents selected from Stage 2 (GS_SSD_STG2_DP versus GS_SSD_STG2), the aggregated TBV variance increased rather than decreased, the percentage of fixed alleles reduced from 0.20 to 0.15%, the genomic relationship reduced from 0.027 to 0.022, and genetic gain reduced from 0.10 to 0.09, after applying the diversity preservation strategy. In the case of parents selected from F_2_ (GS_SSD_F_2__DP versus GS_SSD_F_2_), the aggregated TBV variance loss reduced from 27 to 19, the percentage of alleles fixed reduced from 1.06 to 0.86%, the genomic relationship reduced from 0.117 to 0.045, and genetic gain reduced from 0.35 to 0.15, after applying the diversity preservation strategy. Note that the genetic gain in the F_2_ scenario with diversity preservation was still higher than that of Stage 2 parental selection.

## Discussion

A typical lentil breeding program is comprised of several steps: cross hybridization, seed bulking and generation advance through selfing (to filial generations F_5+_), and field testing for target traits. Field evaluation of breeding progeny usually starts in a single environment with partial replication and eventually moves to multiple environments with full replication later in the breeding cycle (Stages 1 & 2). This information is used in making selections at each of the stages in the breeding cycle. Finally, a subset of best performing breeding lines is selected for final evaluation in National Variety Trials (NVT) for commercialization. The major difference between a conventional phenotypic-based selection and a genomic-assisted selection pipeline is that in PS, breeding selections are made based on phenotypic measurements and in GS cycle, genomic predictions are used for making selections. One breeding cycle is defined as the time taken from crossing to the selection of parents for the next crossing cycle. In the conventional phenotypic scenario of this study, parents for the next breeding cycle are selected based on phenotypic performance in Stage 2. When parents are selected at Stage 2 that amounts to at least 8 years. With the availability of high-density genomic data, the genetic potential of any genotyped plant or line can be accurately estimated with the genomic selection at any stage of the breeding cycle.

### Application of optimal haploid value

The first aim of this simulation was to demonstrate the superiority of genomic selection over phenotypic selection. When parents were selected from Stage 2, genomic selection demonstrated remarkable superiority over phenotypic selection, with an additional 40% benefit achieved when the bulk-up step of F_3_–F_6_ was implemented. This could be due to increased selection intensity at various breeding stages as well as optimizing parental as well as F_1_ intercrosses by choosing the best crosses from all in silico simulated offspring using optimal haploid value (OHV). OHV was first proposed by Daetwyler et al. (2015) to select the best haplotypes and produce elite double haploids in plant breeding, aiming at increasing both genetic gain and genetic diversity. The principle of OHV was applied in the current study by screening all possible crosses that could be generated from primary parents or F_1_s and selecting cross combinations with the best potential combination of haplotypes in the population. While no doubled haploids were simulated, the principle can be applied in breeding programs of inbred crops such as lentil that simply fix lines by selfing. Further, the best 200 F_1_ intercross individuals were selected based on simulating the family mean and standard deviation of GEBVs at F_8_. Without these optimization steps, a genomic selection scheme would likely result in only slightly more genetic gain than phenotypic selection when applied only to select parents at Stage 2.

### Shortening of generation interval

The breeder equation stipulates that genetic change = (genetic standard deviation × selection intensity × selection accuracy)/cycle time. The majority of increases in genetic gain in genomic selection scenarios can be achieved by decreasing cycle time, either by accelerating the generation of inbred lines [field single seed descent (SSD) versus glasshouse accelerated SSD (aSSD)] or by selecting parents earlier from F_6_, F_2_ or even F_1_ rather than from Stage 2. When conducting the bulk-up step (F_3_–F_6_) in a glasshouse (aSSD), the time needed for the bulk-up step was reduced to one and half years from 4 years. The generation interval was reduced from 8.5 to 6 years if parents were selected from Stage 2 and from 5 to 2.5 years if parents were selected from F_6_. When selecting parents from the end of the bulk-up step (F_6_), F_2_ or F_1_, the generation interval was significantly shortened to 5, 1 or 0.5 years, respectively.

Genomic selection has widely been used in livestock and plant breeding to accelerate the rate of genetic improvement. In dairy cattle, the generation interval of the sire of bulls reduced from about 7 years to less than 2.5 years and that of the dam of bulls reduced from about 4 years to nearly 2.5 years after the implementation of genomic selection, and as a result, rates of genetic gain per year increased by 50–100% (García-Ruiz et al. 2016). In forest trees, with the application of genomic selection, the accuracy of GEBVs was lower than that of the estimated breeding values based on pedigree but the generation interval in a forward selection of radiata pine (*Pinus radiata*) was reduced from 17 to 9 years. The genetic gain per year was increased by 21–103% for a trait with low heritability and 22–177% for a trait with high heritability (Li and Dungey 2018). In cereal breeding, new approaches such as speed breeding are being applied to reduce generation intervals and accelerate crop research and breeding. Generation intervals of crops could be shortened further by controlling temperature and applying supplemental lighting in an enclosed growth chamber (Ahmar et al. 2020; Ghosh et al. 2018; Hickey et al. 2019; Jähne et al. 2020; Watson et al. 2018). For example, speed breeding can achieve up to 6 generations per year for spring wheat (*Triticum aestivum*), durum wheat (*T. durum*), barley (*Hordeum vulgare*), 4 generations for chickpea (*Cicer arietinum*), pea (*Pisum sativum*), and canola (*Brassica napus*). Generation interval could be further reduced if the speed breeding and genomic selection are applied together (Hickey et al. 2019; Jighly et al. 2019). The double haploid breeding procedure is another way for shortening the generation interval in some crops such as canola and bread wheat by reducing the time of achieving homozygosity (Daetwyler et al. 2015; Xu et al. 2020).

### Accuracy of GEBV

When parents were selected from F_1_ or F_2_, the accelerating bulk-up step did not affect the generation interval of the population, but it did affect the time of phenotypic performance availability in the training population. As reliable phenotypes of the training population can be only generated in Stage 2, phenotypes for estimating GEBVs were delayed when parents were selected from F_1_, F_2_ and F_6_, which means that the marker effects that were used to calculate GEBVs were all estimated using phenotypic performance at breeding cycle 3. This is the reason why the accuracy of GEBVs decreased from breeding cycle 4 to breeding cycle 8 for the scenarios when parents were selected from F_1_ and F_2_. Although the accuracy of GEBVs declined when no new phenotypes were available, genetic gain per year was still higher in the scenarios with earlier selection than in the scenarios with late selection again highlighting the importance of decreasing cycle time.

### Additional phenotypes adding to the training population

One strategy that was proposed in this study to increase the accuracy of GEBV with an early selection of parents was to obtain phenotypes of certain F_2_ families by planting F_2_s in the field and adding them into the training population. There were new phenotypes for estimating GEBVs at each breeding cycle and the accuracy of GEBVs was quite stable across breeding cycles, and consequently, genetic gain per year increased. Another consequence of phenotyping F_2_ families was the reduction of genetic diversity loss due to less reduction of TBV variances, especially for grain yield. This paper only presented results of an F_2_ phenotyping strategy for the case of parents selected from F_2_. The conclusion of applying this strategy should also be valid for the cases of parents selected from F_6_ and F_1_. With the reduction in the cost of obtaining high-throughput genotypic data, the benefit of applying this strategy will help implement genomic selection in pulses and other plant species with similar breeding habits.

In this strategy, 50 F_2_ families to be phenotyped was determined by testing a range of values with an acceptable GEBV accuracy (shown in Fig. 7). The more F_2_ families are phenotyped the higher accuracy of GEBV that can be achieved. However, increasing F_2_ families may not be feasible due to limitations in the availability of F_2_ seeds and the capacity of phenotyping in the field.

### Strategies of preserving genetic diversity

Genetic diversity is referred to as the variation of heritable characteristics present in a population of the same species. The presence of genetic diversity between individuals within or between species provides an opportunity for breeders to develop new and improved cultivars with desirable characteristics and tolerance to biotic and abiotic stresses. It also facilities crop to have the ability to adapt to varied environments including changing climatic conditions (Bhandari et al. 2017; Govindaraj et al. 2015). Genetic diversity has been measured in three categories of indicators, inbreeding and kinship coefficients (Li et al. 2008; Meuwissen et al. 2020), reduction of the additive genetic variance due to selection and allelic diversity (Bhandari et al. 2017; Doublet et al. 2019). Three genetic diversity measures were used to quantify genetic diversity changes in the current study: the average genomic relationship, the reduction of true breeding value (TBV) variance and the number of alleles fixed among the individuals selected. The average genomic relationship is an indicator of inbreeding level among the germplasms. The higher the average genomic relationship, the higher the inbreeding level and the lower the genetic diversity in the population. The TBV variance is an indicator of the additive genetic variance. The reduction in the additive genetic variance from a selection is often called the Bulmer effect (Bulmer 1971; Falconer and Mackay 1996). The number of alleles fixed describes the number of loci that are fixed across all alleles. The consequences of the increases in the average genomic relationship and the number of alleles fixed and the reduction in true breeding value variance are losses of genetic diversity and, in turn, losses in long-term genetic responses.

Genomic selection has been used to accelerate genetic improvement through increasing the accuracy of estimated breeding values and through shortening the generation interval of the population by early selection. However, genomic selection tends to accelerate the process of loss of genetic diversity in a population, with a consequence of short-term genetic gain and long-term loss (Gibson 1994; Dekkers and Van Arendonk 1998; Jannink 2010; Li et al. 2001). The results in this study showed genomic selection also increased the loss of genetic diversity per unit of time due to the reduction of genetic variation, the loss of favourable QTL alleles and the increase of relatedness between selected individuals. The earlier selection of parents did increase the rate of genetic improvement, but it also increased the rate of genetic diversity loss. The earlier the parents were selected within a breeding cycle, the more genetic diversity was lost. An additional scenario was simulated with five more breeding cycles with PS and GS with parents selected from Stage 2, where GS lost its superiority over PS after seven breeding cycles (Suppl Figure 3). Therefore, strategies of preserving genetic diversity while maximizing genetic response are necessary when implementing genomic selection in plant breeding. In plant and livestock breeding, minimizing the average co-ancestry and inbreeding coefficient, avoiding the selection of closely related individuals, and reducing the loss of favourable alleles are the strategies that are used to preserve genetic diversity. Selection based upon the estimated breeding values tends to select closely related individuals because individuals in a superior family tend to have relatively higher estimated breeding values than those in other inferior families (Daetwyler et al. 2007). One of its consequences is a higher co-ancestry or higher inbreeding coefficient among selected individuals. In genomic selection, the numerator relationship based on pedigree information is replaced by the genomic relationship derived from genetic markers that distribute across the whole genome. Genomic selection can estimate breeding values more accurately than selection based on pedigree information. In the current study, genetic diversity was preserved by applying a penalty on the average genomic relationship and the number of alleles fixed. The rate of genetic diversity loss was significantly reduced by allowing a reduction of genetic gain per unit of time. The values of penalties on the average genomic relationship and the number of alleles fixed were arbitrary. The results showed that preserving genetic diversity in genomic selection is possible and breeders could choose their values to balance the rate of genetic improvement and the rate of genetic diversity loss.

Genetic diversity in a breeding program can also be increased through the incorporation of exotic germplasm from outside of the breeding program; indeed all progressive breeding programs will do this (Matus et al. 2003; Prohens et al. 2017). This impacts genomic selection as the prediction accuracy is often much lower in predicting the performance of germplasm outside of the training population. Furthermore, incorporating exotic germplasm will likely break up productive gene combinations that have been selected over many breeding cycles. Strategies such as backcrossing to elite parents and using genetic information to select for production-related haplotypes before entering phenotypic trials would minimize the effect on prediction accuracy whilst facilitating increased genetic diversity. Such strategies need to be carefully thought through and modelled to design the optimal approach in incorporating this exotic germplasm.

### Costs related to selections

When comparing the benefit of GS over PS, we cannot omit costs related to the selections. The operational and genotyping costs for GS were about AUD$20–30 per sample. However, implementing PS is quite expensive. According to our experience, phenotyping is usually about AUD$50–100 per plot and, in a typical yield assessment trial, each line needs to be evaluated in up to 6 plots. Additional costs of about AUD$20 per line incur for screening disease resistance in pathology trials and of AUD$5 per line for assessing grain quality traits. With GS, we could skip phenotyping for some of the selection candidates; however, in PS every individual/candidate needs to be extensively assessed. With GS we can also make earlier selections in the breeding cycle i.e. F_1_, F_2_ or F_6_ which allows germplasm to be cycled faster in the breeding pipeline, making the potential rate of genetic gain at least doubled or tripled (shown in Fig. 2).

### Simulation

After filtering, there were 63,967 SNPs available of which 30,000 were randomly chosen for simulation. These 30,000 SNPs tag most of the variation spread across the genome in high LD; therefore, a higher number of SNP would not change the results. Reducing the number of SNPs for simulation makes the process faster without compromising analysis.

The aim of the simulations was to provide guidance on polygenic traits at three levels of heritabilities. We simulated grain yield, grain weight and disease resistance as polygenic traits, assuming each being controlled by 1000 QTL. There are two reasons that we chose 1000 QTL for simulating polygenic traits. Firstly, in the literature, a majority of simulation studies from plant and livestock breeding have assumed being controlled by 1000 QTL or less (Brito et al. 2011; Peters et al. 2020; Strandén et al. 2019; Wientjes et al. 2015; Yao et al. 2018; Yin et al. 2014). Secondly, to properly simulate a polygenic trait that are controlled by many small QTL, the number of QTL needs to be greater than the number of independent chromosome segments (*Me*) (Daetwyler et al. 2010). In lentil, due to its limited diversity (Dissanayake et al. 2020; Ferguson et al. 1998; Khazaei et al. 2016), we expect a *Me* of < 1000, and thus we chose 1000 to ensure the trait genetic architecture is truly polygenic.

## Conclusion

GS led to higher genetic gain per unit of time than phenotypic selection by shortening generation intervals. It also led to higher genetic diversity loss per unit of time than phenotypic selection, especially when cycle time was short due to the early selection of parents. The genetic gain from selecting segregating parents early in the breeding cycle at F_1_ or F_2_ stages was substantially higher than selecting later even though prediction accuracy was moderate. There was no advantage to fixing lines for crossing with GS. For a constant number of F_1i_ individuals, increasing the number of F_1i_ families led to lower aggregated genetic gain and less loss of genetic diversity per year. For a constant number of F_1i_ families, increasing the number of seeds from each F_1i_ led to higher aggregated genetic gain and higher loss of genetic diversity per year only when selecting at the F_2_ stage.

Adding additional phenotypes from F_2_ families to the training population increased the accuracy of GEBVs and genetic gain per year and decreased the rate of genetic diversity loss. Genetic diversity was able to be preserved by applying a strategy that restricted the percentage of alleles fixed and the average relationship of parents selected. While this strongly reduced genetic gain for F_2_ selected parents, the gain was still higher than selecting at Stage 2.

## Supplementary Information

Suppl Figure 1. The genetic gain per year achieved in disease resistance, grain weight, grain yield and the aggregated gain when parents selected from STG2 in PS with F_2_ family bulking-up from F_3_ to F_5_ (PS_STG2) and when parents selected from STG2, F_6_, F_2_ and F_1_ in GS with single-seed-descent (SSD) in F_3_–F_6_.

Suppl Figure 2. Reduction in TBV variance per year achieved in disease resistance, grain weight, grain yield and the aggregated gain when parents selected from STG2 in PS with F_2_ family bulking-up from F_3_ to F_5_ (PS_STG2) and when parents selected from STG2, F_6_, F_2_ and F_1_ in GS with single-seed-descent (SSD) in F_3_–F_6_.

Suppl Figure 3. Mean annual aggregated genetic gain when parents selected from STG2 in PS with F_2_ family bulking-up from F_3_ to F_5_ (PS) and in GS with single-seed-descent (GS).

Below is the link to the electronic supplementary material.

Supplementary file1 (TIFF 149 kb)

Supplementary file2 (TIFF 153 kb)

Supplementary file3 (TIFF 75 kb)

## Data Availability

The authors make the input tGBS data available upon request for non-commercial purposes.
