# Supplementary figures and images for: Strategies of preserving genetic diversity while maximizing genetic response from implementing genomic selection in pulse breeding programs
Source: Theor Appl Genet. 2022 Mar 22;135(6):1813–28. doi: 10.1007/s00122-022-04071-6 (PMC9205836; doi:10.1007/s00122-022-04071-6)

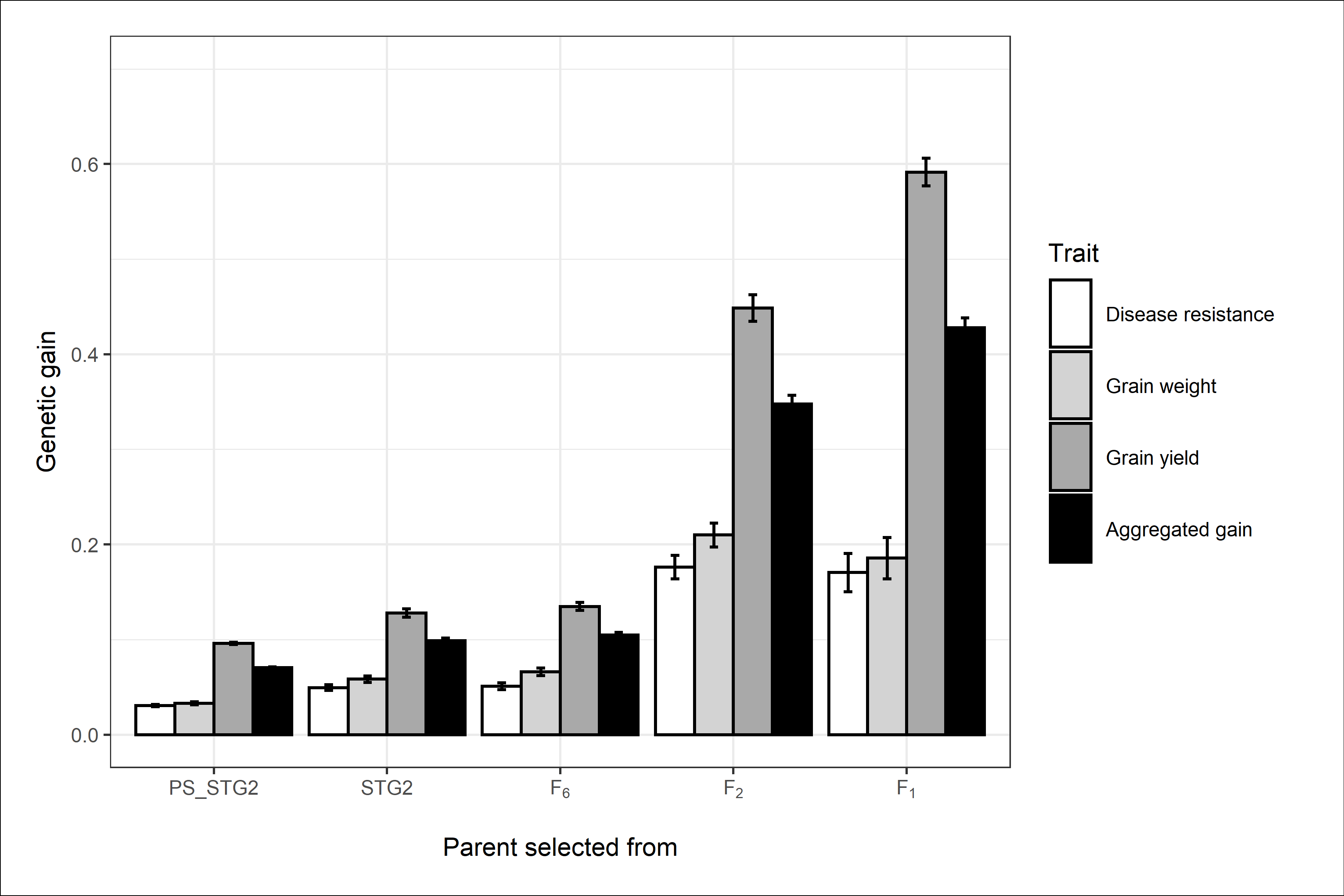

Supplement: Supplementary file 1 — Supplementary file1 (TIFF 149 kb) [file 122_2022_4071_MOESM1_ESM.tiff]

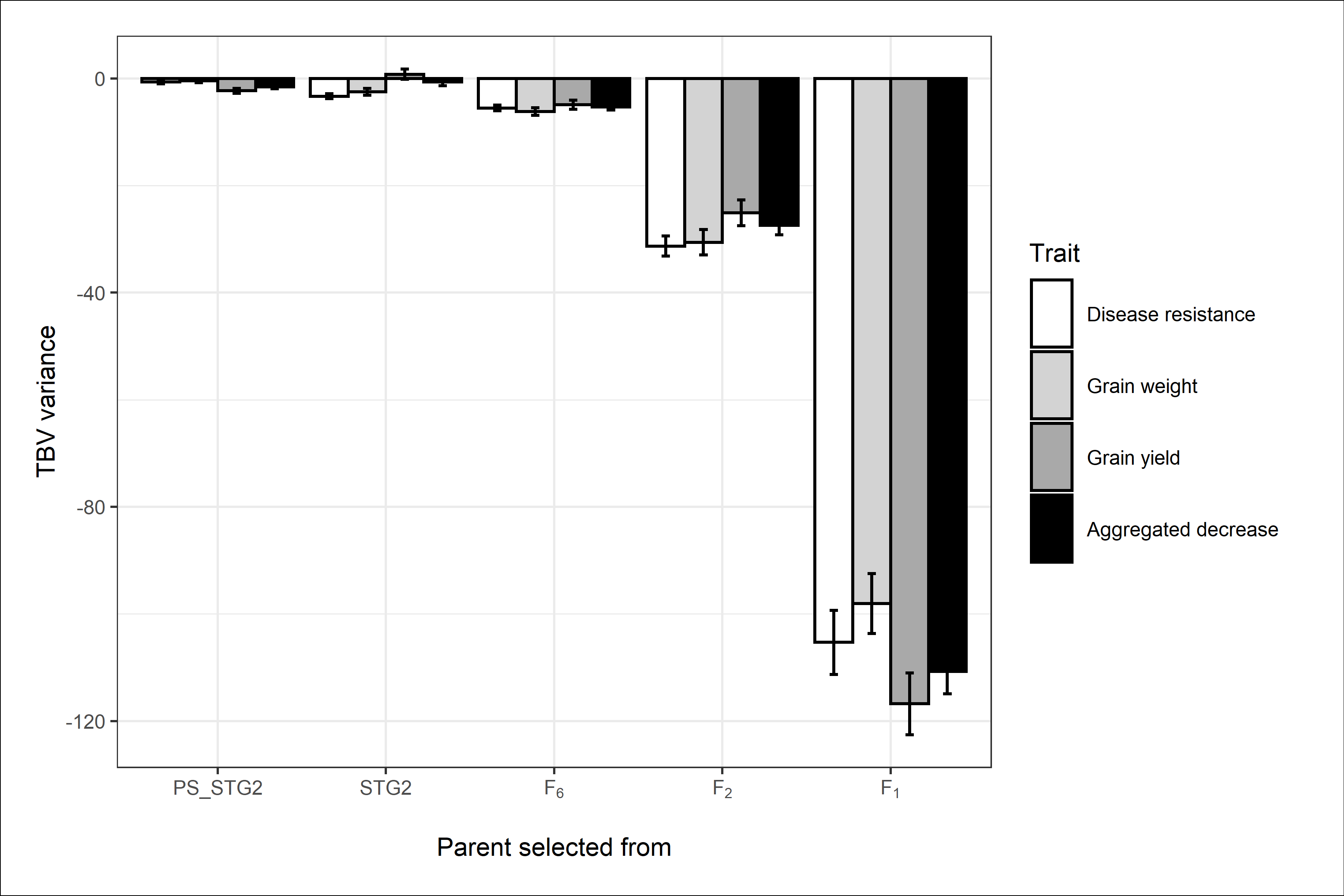

Supplement: Supplementary file 2 — Supplementary file2 (TIFF 153 kb) [file 122_2022_4071_MOESM2_ESM.tiff]

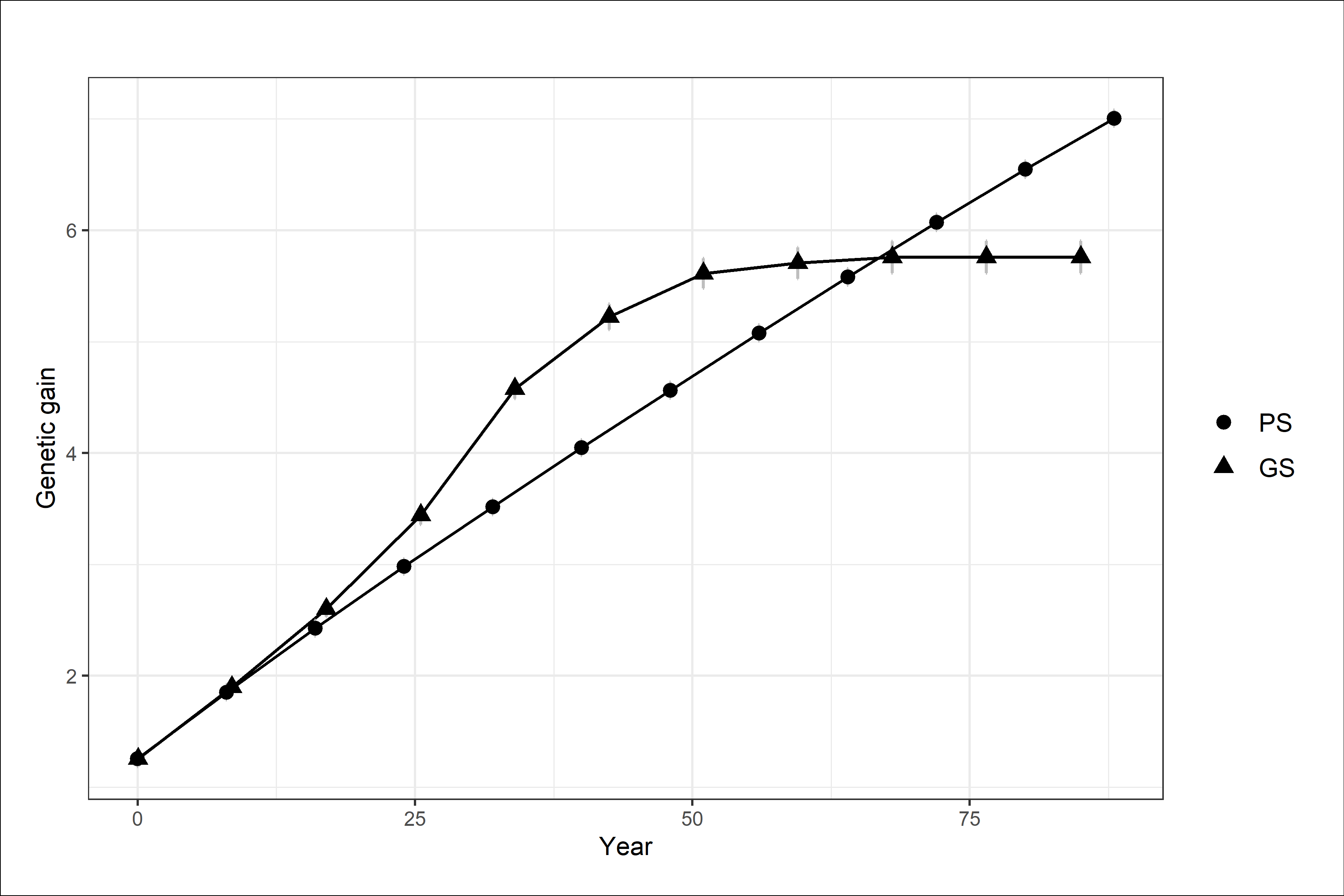

Supplement: Supplementary file 3 — Supplementary file3 (TIFF 75 kb) [file 122_2022_4071_MOESM3_ESM.tiff]
